# Direct medical costs of congenital heart surgery for isolated lesions in Rwanda

**DOI:** 10.1371/journal.pgph.0004462

**Published:** 2025-07-30

**Authors:** Yayehyirad Ejigu, Vongai C. Mlambo, Kara L. Neil, Yihan Lin, Augustin Sendegeya, Stella M. Umuhoza, Josue Murengezi, Corneille Ntihabose

**Affiliations:** 1 King Faisal Hospital Rwanda, Kigali, Rwanda; 2 Stanford University School of Medicine, Palo Alto, California, United States of America; 3 Africa Health Sciences University, Kigali, Rwanda; 4 Department of Cardiothoracic Surgery, Stanford University School of Medicine, Palo Alto, California, United States of America; 5 Department of Health Policy, Economics and Management, University of Rwanda, Kigali, Rwanda; 6 Ministry of Health, Kigali, Rwanda; University of Global Health Equity, RWANDA

## Abstract

Cost transparency is important to facilitate national health infrastructure planning for pediatric cardiac surgery in low-resourced settings. The aim of this paper is to determine direct medical costs of common pediatric congenital heart procedures performed by an in-house cardiac surgery program in Rwanda. Billing information for patients with isolated congenital heart disease who underwent surgery between October 2022 and April 2024 was collected from the hospital management system. Charges were organized into 10 categories, including procedure cost, theater medications and consumables, intensive care unit and ward expenses, anesthesia fees, hospital charges, room charges, testing, and ancillary services. Linear regression was performed to identify perioperative factors associated with increased costs. Costs were converted from Rwandan Francs to US Dollars using the exchange rate 1 USD = 1,262 RWF on 1 January 2024. 117 patients received 8 types of surgeries. Median costs ranged from USD$1,969.04 for patent ductus arteriosus ligation to USD$18,239.00 for arterial switch operation. Excluding the latter, the cost of surgeries was USD$7,662 or less. Theater medications and consumables were the most expensive category accounting for 44.6% (USD$3,071.28) of total costs. This was followed by the cost of cardiothoracic procedure itself which constituted 15.4% (USD$810.90) of total costs. Prolonged hospital stays and operative times increased costs by USD$172.57 and USD$1,015.35 (p < 0.001), respectively. Complications and lesion complexity did not independently predict increased costs (p > 0.05). Direct medical costs of pediatric congenital heart surgeries in Rwanda are generally lower than the mean USD$7,366 plus travel expenses charged abroad. Costs can be lowered by reducing per unit costs of theater consumables and ensuring timely discharge.

## Introduction

Congenital heart disease (CHD) is among the top five non-communicable causes of death and disability in children living in low to middle income countries. Sixty-nine percent of children with CHD die before their first year of life [1]. Timely cardiac surgery can help circumvent this morbid trajectory. Despite this, only 25 centers in sub-Saharan Africa perform pediatric cardiac surgery, leaving 97% of children without the surgical care they need [2].

A systematic review of the national health plans created by African countries in 2016 found no mention of strategies to address the surgical burden of CHD [3]. Part of the challenge in creating sound strategic plans for CHD is the lack of cost transparency regarding the financial resources required to perform surgery. Estimates of USD$4,000 and USD$6,230 for CHD surgery are available in Sudan and Nigeria [4,5]. However, the former does not provide sufficient detail on the cost components included and the latter only estimates costing for atrial septal defect (ASD) repair. A detailed costing analysis for a wide range of procedures would allow an informed comparison of competing alternatives for policymakers.

One such alternative is sending patients abroad for treatment, which is a common practice in many African countries. For example, Nigeria spends approximately USD$1 billion annually sending its citizens abroad for medical care, with one of the top procedures being cardiac related [6]. Prior to establishing an in-house pediatric cardiac surgery program in October 2022, the Government of Rwanda sent patients to Israel, India and Belgium for CHD surgery. For India, medical costs ranged between USD$1,746 – USD$10,700 depending on the procedure. Re-allocating these funds to expand cardiac healthcare infrastructure would ensure that patients have treatment options closer to home that may be cheaper in the long run and more accessible to a wider range of patients [7].

To support decision-making in the creation and implementation of strategic initiatives for CHD surgery, we report the total hospital costs of common congenital heart disease surgeries in Rwanda. Additionally, we identify factors that are associated with increased hospital costs to highlight opportunities for cost savings. Nationally, these results can inform national and institutional healthcare budgets and financing strategies to further develop CHD surgical services. More broadly, this study can foster opportunities for regional collaboration and consolidation of resources to make strides in closing the treatment gap in pediatric cardiac care.

## Methods

### Ethics statements

The study was approved by the Institutional Review Board of King Faisal Hospital Rwanda, and the need for consent was waived (Protocol No. KFH/2024/186/IRB, approved September 21, 2023).

### Study setting

Rwanda’s locally run pediatric cardiac surgery program started in October 2022 and is housed in a quaternary level teaching hospital, King Faisal Hospital Rwanda (KFH) in the capital city, Kigali. As of April 2024, 207 patients with conditions ranging from isolated patent ductus arteriosus (PDA) to transposition of the great arteries (TGA) have undergone surgery.

### Study population

The study population included patients of any age who received congenital cardiac surgery procedures between 8 October 2022 and 15 April 2024 at KFH.

### Data collection

Billing information for the hospital admission was downloaded from the Hospital Information Management System, Napier, and the researchers accessed this data September 25, 2024. We collected the bills for patients who had a single or primary congenital heart disease procedure performed. These procedures included ventricular septal defect (VSD) repair, ASD repair, tetralogy of Fallot (TOF) repair, coarctation of the aorta (COA) repair, PDA ligation, complete atrioventricular canal defect (CAVCD) repair, partial atrioventricular canal defect (PAVCD) repair and arterial switch operation (ASO).

Billing information was condensed into 10 major categories, including theater medications and consumables, the cardiothoracic procedure itself, intensive care unit (ICU) and ward expenses, anesthesia fees, hospital charges, room charges, labs, imaging, bedside procedures and testing, ancillary services and non-cardiac procedures. The amount patients were expected to pay was also recorded. In addition to costing data, key clinical characteristics from the institutional CHD surgical registry were collected to assess whether they are associated with increased expenditure. Variables of interest included age at surgery, sex, insurance, ICU and hospital length of stay (LOS), surgery duration, bypass time, cross clamp time and in-hospital complications.

For insurance, we considered all types of government-sponsored insurance in Rwanda to be public, which includes community-based health insurance, the Rwanda Social Security Board medical scheme for civil servants and employees of private institutions, and the Military Medical Insurance Scheme for military personnel. Although there are other public insurance schemes in Rwanda, these were the only three types of public insurance coverage in our study population. Patients with private insurance schemes were also included in the analysis. All data was collected in Microsoft Excel Version 16.84.

### Data analysis

Data analysis was performed in RStudio (Version 2023.12.1). For baseline characteristics, categorical data was summarized as counts and percentages and numerical data as median with interquartile range. For each type of surgery, the mean and median cost per surgery, median patient payment and median cost per category was calculated. Standard deviation (SD) and interquartile range (IQR) were used to show variability in costs. Using this data, we determined the percentage contribution of each cost category to the total costs. Costs in Rwandan Francs were converted to US dollars using the exchange rate USD$1 = RWF1,262 provided by Exchange-Rates.org on 1 January 2024 [8].

To assess predictors of increased total hospital costs, we performed a univariate and multivariate linear regression. The multivariate linear regression model included variables significant in the univariate analysis using a forward selection approach. Linearity of variables, variance of residuals and normality of residuals were assessed graphically and statistically to ensure that the assumptions of multiple linear regression were met. For all statistical tests the level of significance was set to alpha = 0.05.

## Results

### Patient characteristics

Table 1 shows the characteristics of the 117 patients included in the cost analysis and Table 2 presents the breakdown of clinical characteristics according to surgery type. The median age at surgery was 3.4 years with a slight dominance of female patients at 54% (n = 64). Ninety-four percent (n = 110) patients had some form of public insurance. Repair of simple lesions such as VSD, ASD and PDA accounted for 60% (n = 70) of the procedures. The median ICU and LOS was 2 and 5 days, respectively. Patients who received ASO had the longest ICU and hospital LOS of 11 and 48 days. The overall rate of complications was 19% (n = 22). Complications occurred for all surgery types and were most frequent among patients with TOF (n = 9).

**Table 1:** Study Population Characteristics.

Parameter	Value
Median age at surgery, years (range)	3.41 (0.01 - 20.70)
*Sex, n (%)*	
Male	53 (45)
Female	64 (55)
*Procedure Type, n (%)*	
Simple^*^	70 (60)
Complex	47 (40)
*Insurance Type, n (%)*	
Public	110 (94)
Private	4 (3)
Unknown	3 (3)
*Median Surgery Duration, hours (IQR)*	2.75 (1.5)
*Median Bypass Time, min (IQR)*	92.5 (62.3)
*Median Cross Clamp Time, min (IQR)*	58 (49)
*Median ICU Length of Stay, days (IQR)*	2 (2)
*Median Hospital Length of Stay, days (IQR)*	5 (3)
*Complications, n (%)*	
Yes	22 (19)
No	95 (81)

*Refers to VSD, ASD and PDA repair.

**Table 2:** Patient Characteristics by Surgery Type.

Surgery	No. of Patients	Median age at surgery in years (IQR)	Median ICU LOS in days (IQR)	Median Hospital LOS in days (IQR)	In-Hospital Complications, n (%)
VSD	25	2.32 (3.46)	2 (2)	7 (3)	5 (20)
ASD	14	10.40 (4.92)	2 (0)	5 (1)	2 (14)
TOF	28	4.94 (2.77)	3 (2)	7 (3.25)	9 (33)
COA	13	0.72 (1.97)	2 (0)	5 (2)	1 (9)
PDA	31	1.30 (3.17)	1 (1)	4 (1.75)	1 (3)
CAVCD	4	1.29 (0.86)	8 (3.5)	11 (2.5)	2 (50)
PAVCD	4	10.90 (3.41)	2 (0)	5 (0.5)	1 (25)
ASO	1	10	14	48	1 (100)

### Overall costs and cost contributions

Costs associated with surgeries for each of 8 medical procedures are displayed in Table 3. The median cost per procedure ranged from USD$1,969.04 (±USD$549.92) for PDA repair to USD$18,239.00 for an arterial switch operation (ASO) (Table 3). Excluding ASO, the median cost of surgeries was USD$7,662 or less. The median amount a patient was supposed to contribute to the total hospital cost was zero except for PDA ligation and ASO, where the median payment was USD$107.48 (±USD$265.99) and USD$276.29, respectively.

**Table 3:** Median Charge for Cardiac Surgeries (USD).

Surgery	Mean Charge USD (SD)	Median Charge USD (IQR)	Median Patient Payment USD (IQR)	Percentage of Patients Publicly Insured (%)
VSD (n = 25)	6,565.87 (2,134.91)	6,240.23 (2,363.63)	0.00 (520.20)	92
ASD (n = 14)	4,593.82 (1,199.09)	4,399.58 (1,108.14)	0.00 (380.22)	93
TOF (n = 28)	8,150.77 (3,265.40)	7,263.45 (3,191.93)	0.00 (851.53)	96
COA (n = 13)	2,932.37 (1,472.58)	2,758.62 (1,553.55)	0.00 (0.00)	91
PDA (n = 31)	2,111.67 (886.21)	1,969.04 (549.92)	107.48 (265.99)	97
CAVCD (n = 4)	7,794.66 (1,981.15)	7,662.24 (3,092.37)	0.00 (269.10)	75
PAVCD (n = 4)	7,048.39 (1,143.73)	7,236.39 (1,601.09)	0.00 (1.98)	100
ASO (n = 1)	18,239.00 (N/A)	18,239.00 (N/A)	276.29 (N/A)	100

**Fig 1:**
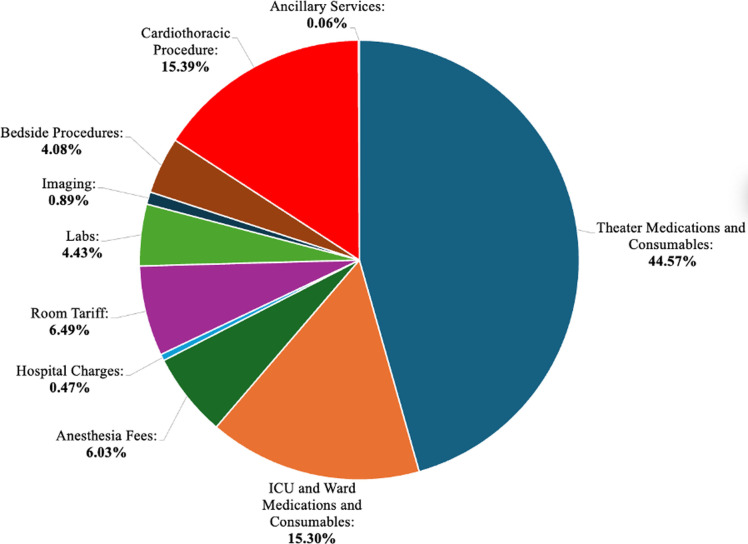
Average Component Cost Contribution for All Procedures.

Across all procedures, the median cost of theater medications and consumables was the most expensive category, accounting for 44.6% (USD$3,071.28) of the total costs. The range was between 22.9% and 52.8% depending on the procedure. This was followed by the cost of the cardiothoracic procedure itself, which was 15.4% (USD$810.90) of the total costs and ICU and ward medications and consumables at 15.3% (USD$712.10). Ancillary services, such as physiotherapy and/or nutrition, contributed minimally to the total costs (Tables 4–5,Fig 1).

**Table 4:** Median Cost of Each Category Per Procedure.

Component	Median Cost (USD)								
	VSD	ASD	TOF	COA	PDA	CAVCD	PAVCD	ASO	All
Theater Medications and Consumables	3,014.30	2,241.10	3,603.53	632.24	493.26	3,128.26	3,818.56	6,255.04	3,071.28
Cardiothoracic Procedure	633.91	507.13	987.89	507.13	316.96	1,249.29	1,177.63	2,678.05	810.90
ICU and Ward Medications and Consumables	702.55	285.58	721.65	618.39	396.82	1,430.81	863.12	4,742.73	712.10
Anesthesia Fees	253.57	240.69	397.99	211.97	138.67	532.48	476.62	952.71	325.78
Hospital Charges	27.73	15.85	35.66	15.85	15.85	46.55	28.72	55.47	28.23
Room Tariff	297.15	173.77	412.76	203.13	143.70	705.90	147.84	1,604.60	250.14
Labs	188.37	155.79	201.07	149.19	104.56	543.72	136.30	1,145.86	172.08
Imaging	35.66	23.77	35.66	35.66	23.77	118.86	26.74	229.79	35.66
Bedside Procedures and Testing	239.55	294.61	313.55	162.16	109.56	283.07	105.57	471.76	261.31
Other Procedures	0.00	0.00	0.00	0.00	0.00	0.00	0.00	63.39	0.00
Ancillary Services	4.95	2.48	4.95	0.00	0.00	25.75	2.48	39.62	3.71

**Table 5:** Component Contribution to Total Cost Per Procedure.

Component	Percentage of Total Cost (%)								
	VSD	ASD	TOF	COA	PDA	CAVCD	PAVCD	ASO	All
Theater Medications and Consumables	48.30	50.94	49.61	22.92	25.05	40.83	52.77	34.29	44.57
Cardiothoracic Procedure	10.16	11.53	13.60	18.38	16.10	16.30	16.27	14.68	15.39
ICU and Ward Medications and Consumables	11.26	6.49	9.94	22.42	20.15	18.67	11.93	26.00	15.30
Anesthesia Fees	4.06	5.47	5.48	7.68	7.04	6.95	6.59	5.22	6.03
Hospital Charges	0.44	0.36	0.49	0.57	0.80	0.61	0.40	0.30	0.47
Room Tariff	4.76	3.95	5.68	7.36	7.30	9.21	2.04	8.80	6.49
Labs	3.02	3.54	2.77	5.41	5.31	7.10	1.88	6.28	4.43
Imaging	0.57	0.54	0.49	1.29	1.21	1.55	0.37	1.26	0.89
Bedside Procedures and Testing	3.84	6.70	4.32	5.88	5.56	3.69	1.46	2.59	4.08
Other Procedures	0.00	0.00	0.00	0.00	0.00	0.00	0.00	0.35	0.00
Ancillary Services	0.08	0.06	0.07	0.00	0.00	0.34	0.03	0.22	0.06

### Predictors of increased costs

In the univariate analysis, in-hospital complications, complexity of lesion, length of stay, surgery duration, bypass time and cross clamp time were significantly associated with increased total hospital costs (p < 0.001) (Table 6). Since surgery duration, bypass time and cross clamp time were significantly correlated with each other (r = 0.79 - 0.89, p < 0.001), we applied principal component analysis to create a linear combination of these variables which was subsequently used in the multivariate analysis.

**Table 6:** Regression Analysis for Total Hospital Costs.

Parameter	Bivariate			Multivariable		
	Estimate	95% CI	P value	Estimate	95% CI	P Value
*Age*	76.03	(-60.05) - 212.12	0.271			
*Sex*						
Female	Reference					
Male	738.32	(-497.68) – 1,974.32	0.239			
*Complication*						
No	Reference					
Yes	3,474.54	2,026.20 – 4,922.88	<0.001^*^	-169.58	(-1,108.69) – 769.53	0.721
*Surgery Complexity*						
Simple	Reference					
Complex	2,821.07	1,670.99 – 3,971.16	<0.001^*^	380.81	(-124.61) - 1,204.38	0.270
ICU Length of Stay	514.84	406.62 - 623.06	<0.001^*^	119.56	(-3.75) - 253.55	0.067
Hospital Length of Stay	388.42	317.29 - 459.55	<0.001^*^	172.57	82.77 – 256.40	<0.001^*^
Surgery Duration	1,480.76	1,147.59 - 1,813.93	<0.001^*^			
Bypass Time	30.37	17.42 - 43.33	<0.001^*^			
Cross Clamp Time	45.30	30.73 – 59.87	<0.001^*^			
Surgery Duration + Cross Clamp + Bypass	1,478.19	1,220.73 – 1,735.65	<0.001^*^	1,015.35	751.37 – 1,219.97	<0.001^*^

*Indicates significant variables at alpha = 0.05.

The multivariable linear regression was significant and explained 77% of the variability in costs (Adjusted R-squared: 0.77, F statistic 72.56 on 5 and 102 DF, AIC 1894, p value < 0.001). Only prolonged hospital length of stay and the linear combination of operative times was significantly associated with increased hospital costs. Each additional hospital day increased costs by USD$172.57 (p < 0.001) and longer operative times increased costs by USD$1,015.35 (p < 0.001). Each additional day in the ICU was associated with an additional USD$119.56; however, this did not reach the level of significance (p = 0.067). Notably, complications and lesion complexity on their own did not predict increased hospital costs.

## Discussion

We present an in-depth cost analysis for a wide range of pediatric cardiac surgery procedures performed by a locally run surgery program in sub-Saharan Africa. Total median hospital costs for the surgery admission were as low as USD$1,969.04 for PDA repair, and 7 out of the 8 surgeries had median costs less than USD$7,700. Local direct medical costs are less expensive compared to the average of USD$7,366 per patient plus travel expenses for treatment abroad. The largest contributor to these costs was theater medications and consumables, and costs increased with prolonged hospital length of stay and longer operative times.

### Global comparison

As expected, the cost of pediatric cardiac surgery in Rwanda is significantly lower compared to high income countries. In a United States nationwide cost analysis, the hospital admission charges for pediatric cardiac surgery procedures ranged between USD$32,088 - USD$100,058 [9]. However, when compared to a country with universal health insurance like Belgium, the difference was not as extreme. For example, the mean cost of an ASD and VSD repair was USD$11,683.11 and USD$14,514.00 compared to USD$4,593.82 (±1,199.09) and USD$6,565.87 (±2,134.91) in this study, respectively [10]. Importantly, the direct medical costs of surgery are less expensive than the total costs of treatment abroad, reinforcing the long-term economic benefits of a local pediatric cardiac surgery program in our setting.

Direct medical costs in this study are also less expensive than in most other limited resourced countries. In Nigeria, ASD repair was USD$6,230, and in Lebanon, the mean bill for CHD surgery was USD$27,450 (±$13,685) per patient [5,11]. However, Lebanon performs a higher case load of complex surgeries that may drive up average costs. One reason for the lower figures reported in this study may be due to incomplete accounting for the cost of labor. The only personnel whose time was explicitly billed for was anesthesia services. It is possible that labor costs of other personnel are implicitly integrated into cost categories. For example, operating room staff services may have been included in the cost of the cardiothoracic procedure since other theater expenses are separate. Notably, the Nigerian study also did not include all personnel costs [5]. Clear costing for the workforce involved in providing cardiac surgery in sub-Saharan Africa is important for planning as these expenses often make up a significant percentage of total medical costs [12].

A country that is well-known to provide surgical procedures at extremely low costs is India. One center is able to provide surgeries like ASD, VSD and TOF repair for less than USD$2,000 [13]. This is facilitated by a thriving local manufacturing industry in India that produces low-cost cardiac surgery equipment and devices. Additionally, India performs approximately 27,000 surgeries annually, allowing them to benefit from economies of scale that enable bulk purchase of supplies and efficiently distribute fixed costs [13,14]. As case volume increases over time, the program will also benefit from cost reductions.

### Ability to pay for pediatric cardiac surgery

For all procedures except PDA repair and ASO, the median patient co-payment was zero, which means many patients were receiving their surgery at no cost. Typically, on public insurance, patients are expected to pay 10% of the total costs as a co-payment. However, the GDP per capita in 2022 according to the World Bank was USD$966.20, which means it is often not possible for families to cover this fee without significant economic consequences for their household [15]. KFH has a philanthropic arm that mobilizes funds from private donors to support families who cannot pay the 10% contribution. As the program grows and treats more patients, it may not be sustainable to continue absorbing these costs, as donor revenue may become insufficient or unreliable [13]. One way to address this is to add new income streams by attracting paying patients in the region either for cardiac surgery or other specialist services uniquely available at KFH. For example, KFH has one of the few catheterization labs in sub-Saharan Africa that provides otherwise limited minimally invasive cardiac and interventional radiology care. The funds from these patients could then be used to support low-income patients [16]. Finding ways to reduce costs can also help stretch existing institutional and national budgets to ensure they are sufficient in the long term. Such cost-saving strategies are discussed in the next section.

### Cost-saving opportunities

Theater medications and consumables accounted for almost half of the total hospital costs. This is surprising given that patients are in the operating room for a median of 3.6 hours compared to 7 days in the ICU and wards. One reason for this finding is that the accounting for theater consumables involves charges for special equipment that is not routinely used in the ICU or ward. For example, a patient undergoing a VSD repair was charged for use of individual cardiopulmonary bypass machine components in the operating room. Collectively, these components can add up to a sizable sum. Additionally, the operating room is a more controlled environment that facilitates meticulous recording of each material used. In the ICU and ward, this detailed documentation is not currently feasible, possibly resulting in less accurate accounting. As such, a digitalized way of collecting accurate payments from all ICU medications, procedures and staff payments may help to strengthen this.

In the Nigerian study, theater expenses for ASD repair were also the majority, accounting for 39% of total costs, suggesting that the operating room is a significant source of resource use [5]. Negotiating or using less expensive versions of expensive theater consumables can help decrease the cost of surgery. Collaboration among centers performing pediatric heart surgeries in Rwanda, Tanzania, Uganda and Kenya can reduce per unit costs of theater medications and consumables through direct pooled procurement.

The regression analysis suggests that another way to decrease total hospital costs is to address hospital length of stay, which was demonstrated in the US and Belgian cost studies as well [9,10]. Hospital length of stay can be prolonged due to medical or logistical factors. From a medical perspective, complications should be promptly diagnosed and addressed to prevent prolongation of admission. In our setting this is most commonly pneumonia. From a logistical perspective, delayed discharge is a global problem and not only increases costs but also heightens the risk of infections and prolonged rehabilitation [17]. However, the median hospital length of stay in this cohort was 5 days which is low compared to other centers, suggesting logistical factors are not contributory for most patients [18–20]. In some cases, there may be hesitancy to discharge patients especially given that most of them live in rural Rwanda with limited access to specialists if an adverse event were to occur [21]. Clear discharge criteria and emergency contingency plans can help circumvent this issue to enable safe and timely discharge.

### Limitations

This study’s limitations stem primarily from the use of billing data, which was not always available for every eligible patient. Nonetheless, we obtained billing information for a sufficient number of patients per surgery, allowing reliable cost analysis, except for ASO, where only one bill was available, precluding cost variation assessment. The billing data included charges for anesthesiologist fees and post-operative specialist care during hospital stays but omitted explicit fees for cardiothoracic surgeons, operating room staff, and ICU staff, potentially underestimating total costs. However, even with the addition of staff compensation, costs will likely still be comparable to peers in low-to-middle income settings and less expensive than treatment abroad due to the relatively lower cost of labor in Rwanda. While the figures represent institutional charges rather than the true cost of surgery, they provide a useful benchmark for healthcare planning, with time-driven activity-based costing suggested for more precise cost evaluations. Additionally, non-medical patient costs, such as transportation, food, and caregiver productivity losses, were not accounted for in this analysis. Although the study does not explicitly account for labor costs, these are typically a small proportion of total costs. There are no known published salary estimates for staff required for congenital heart surgery in Rwanda. As such, even though our analysis excluded labor, it is not a major cost category in our setting. However, future time-based costing analyses of personnel involvement in the care of cardiac surgical patients in Rwanda would be informative.

Another limitation is the lack of consideration for donated equipment and materials, which were significant during the program’s early stages but are now less frequent and unlikely to affect current cost estimates. The role of the national insurance coverage is primarily patient-facing, covering 85–90% of costs for publicly insured patients, with subsidies for those unable to afford their contribution. These subsidies do not impact hospital-incurred costs. Furthermore, while consumables incur a 60% markup, imaging, lab tests, and procedures do not, suggesting the provided costs may slightly overestimate actual patient expenses and represent an upper bound for this setting.

## Conclusion

The costs of providing pediatric cardiac surgery in a locally run program in Rwanda are low compared to other countries and more affordable than sending patients abroad. This motivates continued national investment into the program to intervene in avoidable deaths and reduce exorbitant expenditures associated with medical tourism. Costs can be further lowered by ensuring timely discharge and reducing the cost of theater consumables by establishing procurement collectives with other cardiac centers to benefit from economies of scale. In this way, children in Rwanda and the region will continue to have affordable life-saving cardiac care close to home.

## Supporting information

S1 Data Dataset. (XLSX)
